# Supplementary material for: Possible Roles of Transition Metal Cations in the Formation of Interstellar Benzene via Catalytic Acetylene Cyclotrimerization
Source: Molecules. 2023 Nov 6;28(21):7454. doi: 10.3390/molecules28217454 (PMC10649463; doi:10.3390/molecules28217454)
Supplement: Supplementary file 1 [file molecules-28-07454-s001.zip › molecules-2685804-supplementary/supplement_proof/supplement_proof.pdf]

Possible Roles of Transition Metal Cations in the Formation of Interstellar Benzene via Catalytic Acetylene Cyclotrimerization

Tatsuhiro Murakami [1,2,*], Naoki Matsumoto [1], Takashi Fujihara [1,3] and Toshiyuki Takayanagi [1]

[1] Department of Chemistry, Saitama University, Shimo-Okubo 255, Sakura-ku, Saitama City, Saitama, 338-8570, Japan
[2] Department of Materials & Life Sciences, Faculty of Science & Technology, Sophia University, 7-1 Kioicho, Chiyoda-ku, Tokyo, 102-8554, Japan
[3] Comprehensive Analysis Center for Science, Saitama University, Shimo-Okubo 255, Sakura-ku, Saitama City, Saitama, 338-8570, Japan
* Correspondence: murakamit@mail.saitama-u.ac.jp; Tel.: +81-48-858-9113 (T.M.)

Supplementary Material

Computational Details

The UCCSD(T)/6-311G(d) implemented in Gaussian09 and the explicitly correlated local coupled-cluster with pair natural orbital (PNO-LCCSD(T)-F12) [64,65] calculations using the cc-pVTZ basis set implemented in Molpro 2022 [66] were performed to compare the potential barrier height from the DFT-D3 results with those obtained at the CCSD(T)-level. Moreover, 2-state averaged complete active space second-order perturbation theory using the density fitting (DF-CASPT2) method [67] with the cc-pVTZ basis set implemented in Molpro 2022 was employed to consider the multireference behavior in the transition between *d* orbitals (See Table S4).

Table. S1. Relative Energies (in kcal/mol) Calculated Using B3LYP-D3(BJ)/def2-TZVP at the Benzene-Transition-Metal (benzene-TM) Complex and the Product Fragments (Benzene + TM) for the Neutral Sc and Ti-Catalyst Systems. The Zero Energy is Defined as the Energy Level of the Reactants containing Metal Cations.

	(benzene-TM) complex	benzene + TM
Sc(0)		
Quartet	-233.0 (-7.3)	-207.5 (32.8)
Doublet	-240.3 (0.0)	-228.6 (11.7)
Ti(0)		
Quintet	-238.3 (-16.2)	-221.4 (0.7)
Triplet	-248.7 (-26.6)	-221.6 (0.5)
Singlet	-222.1 (0.0)	-187.1 (35.0)

Table. S2. Vertical Excitation Energies (in kcal/mol) at the Stationary Points for ScNC(C_6H_6) and TiNC(C_6H_6) Calculated Using B3LYP-D3(BJ)/def2-TZVP.

	Reactant	INT1	TS1	INT2	TS2	PC	Product
ScNC-C_6H_6							
Quintet	155.2	127.9	124.1	85.7	82.0	33.9	60.3
Triplet	66.9	49.6	48.0	9.6	1.8	-71.7	-37.3
Singlet	0.0	-16.9	-14.0	-57.1	-57.0	-91.2	-38.1
TiNC-C_6H_6							
Sextet	155.2	95.7	87.4	72.3	65.8	0.4	17.6
Quartet	44.7	15.9	13.0	-10.4	-19.2	-109.7	-63.6
Doublet	0.0	-48.9	-46.7	-79.3	-78.7	-119.4	-50.2

Table. S3. Relative Energies (in kcal/mol) at the Stationary Points for the $^{1}Sc^{+}(C_6H_6)$, $^{2}Ti^{+}(C_6H_6)$, $^{1}ScNC(C_6H_6)$ and $^{2}TiNC(C_6H_6)$ calculated by B3LYP-D3(BJ)/def2-TZVP, B3LYP-D3(BJ)/def2-SVPP, and PNO-LCCSD(T)-F12/cc-PVTZ. The PNO-LCCSD(T)-F12/cc-pVTZ and DF-CASPT2/cc-pVTZ. Calculations were Performed at the B3LYP-D3(BJ)/def2-TZVP Geometries for the $^{1}Sc^{+}(C_6H_6)$ and $^{2}Ti^{+}(C_6H_6)$, and at the B3LYP-D3(BJ)/def2-SVPP Geometries for $^{1}ScNC(C_6H_6)$ and $^{2}TiNC(C_6H_6)$.

	B3LYP-D3(BJ)/ def2-TZVP	B3LYP-D3(BJ)/ def2-SVPP	PNO-LCCSD(T)- F12/cc-pVTZ	DF-CASPT2/ cc-pVTZ
$^{1}Sc^{+}$-C_6H_6				CAS(6e,8o)
Reactant	0.0	---	0.0	0.0
INT1	-59.2 (0.0)	---	-56.5 (0.0)	-50.0(0.0)
TS1	-56.8 (2.4)	---	-51.8 (4.7)	-36.3(13.7)
PC	-133.0	---	-131.7	-112.5
$^{2}Ti^{+}$-C_6H_6				CAS(7e,8o)
Reactant	0.0	---	0.0	0.0
INT1	-55.3 (0.0)	---	-51.7 (0.0)	-45.6(0.0)
TS1	-47.9 (7.4)	---	-42.9 (8.8)	-26.4(19.2)
PC	-126.9	---	-121.3	-128.8
$^{1}ScNC$-C_6H_6				CAS(6e,8o)
Reactant	0.0	0.0	0.0	0.0
INT1	-16.9 (0.0)	-18.3 (0.0)	-17.0 (0.0)	-17.1(0.0)
TS1	-14.0 (2.9)	-16.0 (2.3)	-9.7 (7.3)	-2.0(15.1)
INT2	-57.1	---	---	---
TS2	-57.0	---	---	---
PC	-91.2	-98.3	-89.1	-68.9
$^{2}TiNC$-C_6H_6				CAS(9e,9o)
Reactant	0.0	0.0	0.0	0.0
INT1	-48.9 (0.0)	-55.7 (0.0)	-44.8 (0.0)	-52.8(0.0)
TS1	-46.7 (2.2)	-53.7 (2.0)	-41.9 (2.9)	-37.9(14.9)
INT2	-79.3	-90.1	---	---
TS2	-78.7	-89.8	---	---
PC	-119.4	-131.0	-125.7	-124.5

Table. S4. Relative Energies (in kcal/mol) at the Stationary Points for the $^1Sc^+(C_6H_6)$, $^2Ti^+(C_6H_6)$, $^1ScNC(C_6H_6)$ and $^2TiNC(C_6H_6)$ calculated by 2-state averaged DF-CASPT2/cc-pVTZ.

	Reactant	INT1	TS1	PC
		Sc^+-C_6H_6, CAS(6e,8o)		
S_1	52.9	28.7	45.0	-77.9
S_0	0.0	-50.0	-36.3	-112.5
		Ti^+-C_6H_6, CAS(7e,8o)		
D_1	47.8	19.0	-18.8	-90.5
D_0	0.0	-45.6	-26.4	-128.8
		ScNC-C_6H_6, CAS(6e,8o)		
S_1	84.4	82.1	93.1	-39.9
S_0	0.0	-17.1	-2.0	-68.9
		TiNC-C_6H_6, CAS(9e,9o)		
D_1	34.9	5.7	8.4	-109.7
D_0	0.0	-52.8	-37.9	-124.5

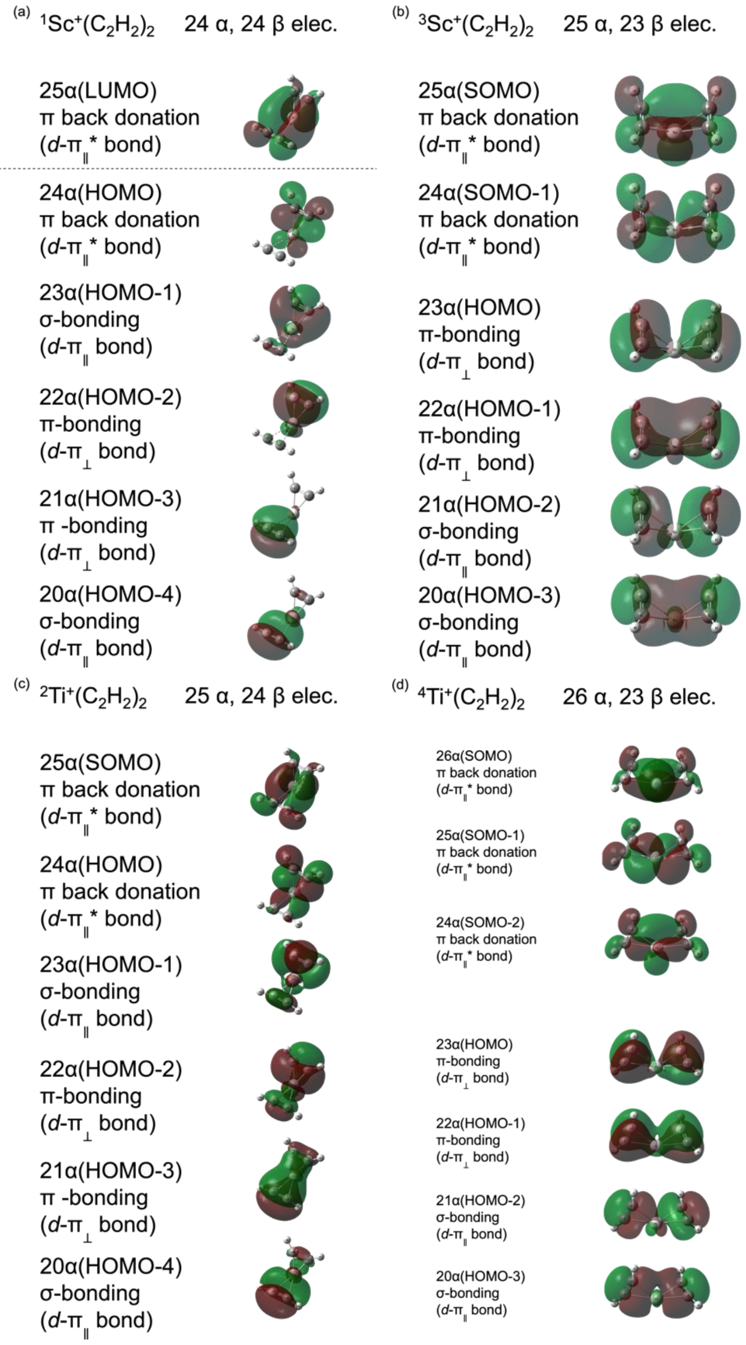

Figure S1. Molecular orbitals contributing to d-$\pi_{\parallel}$ and d-$\pi_{\perp}$ interactions and π-back-donation for (a) singlet $Sc^+(C_2H_2)_2$, (b) triplet $Sc^+(C_2H_2)_2$, (c) doublet $Ti^+(C_2H_2)_2$, and (d) quartet $Ti^+(C_2H_2)_2$ calculated using B3LYP-D3(BJ)/def2-TZVP

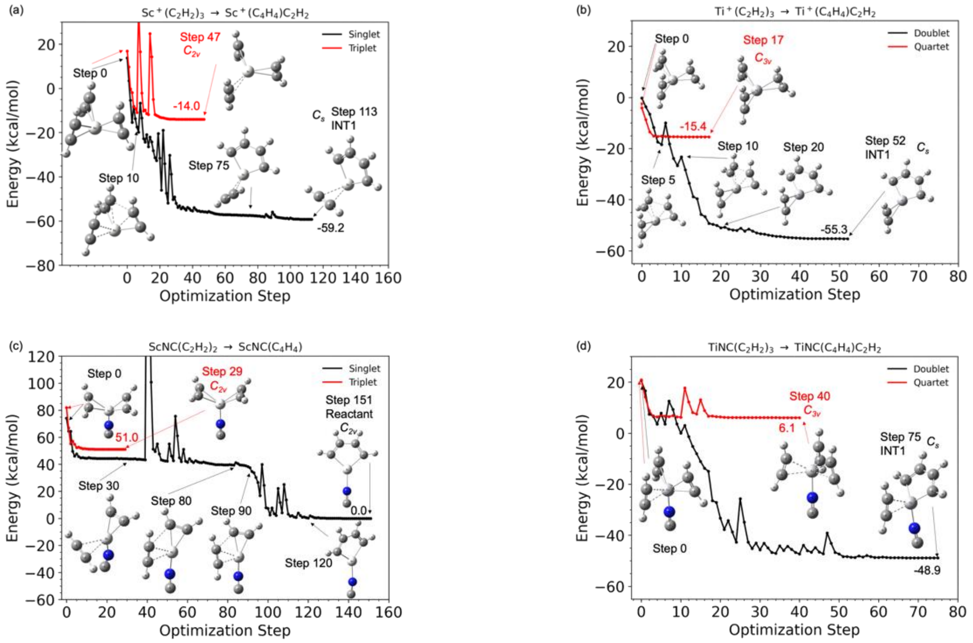

Figure S2. Potential energy minimization profiles calculated using B3LYP-D3(BJ)/def2-TZVP for (a) $Sc^+(C_2H_2)_3$ of singlet (black line) and triplet (red line) states, (b) $Ti^+(C_2H_2)_3$ of doublet (black) and quartet (red), (c) $ScNC(C_2H_2)_2$ of singlet (black) and triplet (red), and (d) $TiNC(C_2H_2)_3$ of doublet (black) and quartet (red), with the molecular structures. The optimized energies (in kcal/mol) and those symmetries are written in each figure. The potential energies for the reactants are set as 0 kcal/mol.

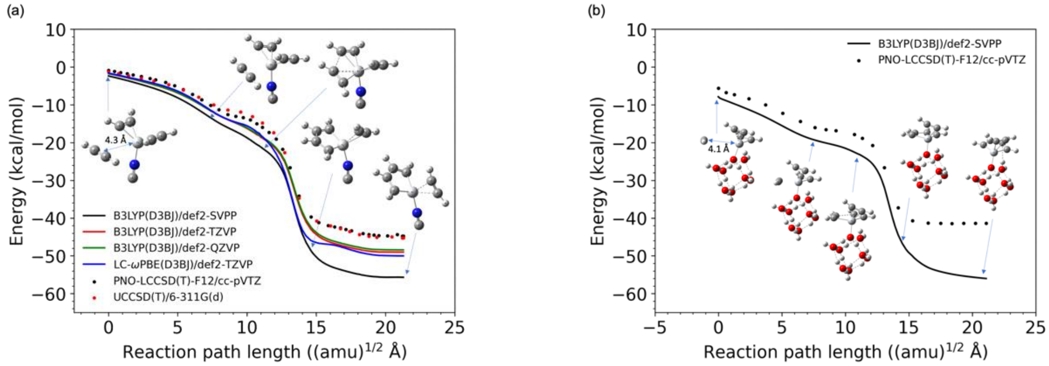

Figure S3. (a) Minimum energy paths (MEPs) for the doublet $TiNC(C_2H_2)_2$ with isolated acetylene; black, red, green, and blue lines, and black and red dots represent the calculations at the B3LYP-D3(BJ)/ def2-SVPP, B3LYP-D3(BJ)/ def2-TZVP, B3LYP-D3(BJ)/ def2-QZVP, LC-ωPBE-D3(BJ)/ def2-TZVP, and PNO-LCCSD(T)-F12/ cc-pVTZ and UCCSD(T)/ 6-311G(d) levels; (b) MEPs of doublet $Ti^+(H_2O)_8(C_2H_2)_2$ with isolated acetylene; black line and dots represent calculations at the B3LYP-D3(BJ)/ def2-SVPP and PNO-LCCSD(T)-F12/ cc-pVTZ levels.

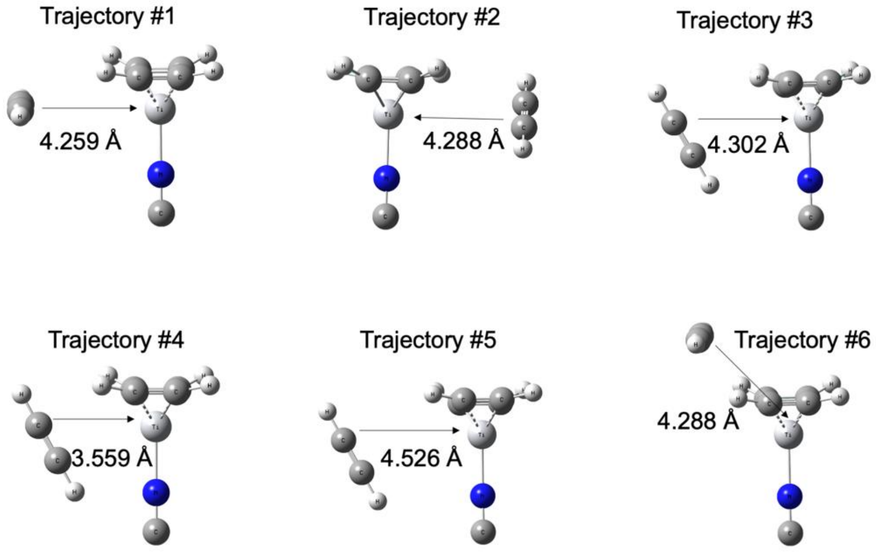

Figure S4. Initial configurations of the $TiNC(C_2H_2)_3$ for molecular dynamics

Cartesian coordinates for all the stationary points on the potential energy surfaces obtained from the UB3LYP/def2-TZVP (GD3BJ dispersion, int=ultrafine) calculations.

$(C_2H_2)_2$-Sc^+ reactant

Sc	0.097474288682	-2.205311655782	0.959971762699
C	1.233247522190	0.058273724648	1.776837793411
C	-1.822905288155	-2.677396896215	1.371009480728
C	-1.048584707504	-3.075506831325	2.377355205909
C	0.533249008704	0.418151872323	0.866576496318
H	1.860108022735	-0.180964244123	2.610867547764
H	-2.895425706942	-2.740660888374	1.220062763119
H	-1.283991642628	-3.569162298679	3.314359533995
H	-0.081356087082	0.817106497528	0.086242886058

ENERGY = -915.255461876000

$(C_2H_2)_3$-Sc^+ INT1

Sc	-0.206756658393	-1.770025575715	0.266380687999
C	0.830438703292	-0.034815187231	0.664603847285
C	-1.185604547215	0.315112442052	-0.849233975606
C	-0.936870477431	-1.992995470830	2.742689809396
C	-1.911519046704	-2.348605418795	2.130111320677
C	0.020754144955	0.754056472984	-0.090982140731
C	-1.681937798607	-0.949062515940	-0.914845395194
H	1.678906061440	0.413832428305	1.168632063843
H	-1.669420617724	1.131304426427	-1.382036291216
H	-0.115686345578	-1.676688920276	3.349662573224
H	-2.815496785550	-2.661680138951	1.653010895519
H	0.220163077726	1.818780931792	-0.194536344602
H	-2.572962119865	-1.133377780210	-1.504189194562

ENERGY = -992.716588706000
ZPVE = 0.090452944110

$(C_2H_2)_3$-Sc^+ TS1

Sc	-0.062474782280	-1.768449197184	0.275330778529
C	0.856374595403	0.034138012570	0.690313767972
C	-1.262915840114	0.129474641713	-0.730939211514
C	-0.976197186398	-2.132637475247	2.385923469411
C	-2.050971925174	-1.907188369972	1.847610181232
C	-0.040548485539	0.695673954947	-0.080542260334
C	-1.860525018488	-1.062910374280	-0.473064584023
H	1.680292177566	0.579511869812	1.133645182631
H	-1.691729342782	0.801752265873	-1.470955026111
H	-0.293853118626	-2.370633894228	3.178644155084
H	-3.079789236981	-1.779878668519	1.594709637141
H	0.083456438539	1.752685644786	-0.306476618102
H	-2.812091985126	-1.314706190276	-0.925764131914

ENERGY = -992.712793628000

ZPVE = 0.090666861636

(C_6H_6)-Sc^+ Product

Sc	0.054451903782	-1.950497327073	0.031073149588
C	0.265170655219	-0.222510042156	1.375035121244
C	-1.269343852992	-0.043884069116	-0.530543497133
C	-0.745840803821	-1.001085992363	2.070932124817
C	-1.847410132145	-1.469288105544	1.392836115226
C	-0.167797257591	0.424345854372	0.147570742974
C	-2.007976505407	-1.188721943767	-0.024225790739
H	1.102741559026	0.179181654172	1.924437823339
H	-1.559486669607	0.402014981362	-1.472383843067
H	-0.597970526117	-1.255678626688	3.111791841098
H	-2.572032686480	-2.094749539595	1.896621298384
H	0.414552607224	1.241113012446	-0.257195831751
H	-2.897131050275	-1.521056865098	-0.537723285424

ENERGY = -992.834191153000

ZPVE = 0.100071499038

$(C_2H_2)_2$-Ti^+ reactant

Ti	0.177285724764	-2.144402634491	0.922796115781
C	1.048497735297	-0.230652703652	1.816399941952
C	-1.698251413067	-2.512179839088	1.352179065275
C	-0.934418925476	-2.904757090478	2.345198879580
C	0.338048482888	0.134447228248	0.892999003397
H	1.698526786872	-0.322273847484	2.665006672270
H	-2.753339241573	-2.485674857637	1.110881690566
H	-1.053634678313	-3.359240078851	3.320580922448
H	-0.230899061389	0.669263103432	0.157241178732

ENERGY = -1003.995990090000

$(C_2H_2)_3$-Ti^+ INT1

Ti	-0.497263490978	-1.685279133525	-0.518784864088
C	0.328231007018	0.005129336000	-1.129934059274
C	-0.743094222149	0.024878890587	1.184582071152
C	-2.162879385484	-2.202177447319	-1.971467027559
C	-1.429180498512	-3.180419416535	-1.950994110468
C	0.061763613113	0.604749728406	0.055477774157
C	-1.335471971610	-1.193507782080	1.204001266567
H	0.861343375636	0.509951661296	-1.925033687372
H	-0.832631323013	0.708938611373	2.025175617303
H	-2.921189940051	-1.461118017207	-2.131338488281
H	-0.986055833194	-4.142244394998	-2.129174730908
H	0.417255681646	1.609438443722	0.271760418086
H	-1.956472338200	-1.520173857246	2.027946667757

ENERGY = -1081.450802790000

ZPVE = 0.090482171252

$(C_2H_2)_3$-Ti^+ TS1

Ti	-0.282729126217	-1.910814398853	-0.399406976048
C	-0.083922792355	-0.148859351942	-1.295480070088
C	-0.953244561810	0.061570943803	1.049583314353
C	-1.788841340048	-1.578997599169	-2.059824747610
C	-1.643898579034	-2.782046266675	-1.765179015473
C	-0.327922857431	0.590699555703	-0.168932094693
C	-1.219434423918	-1.258282513077	1.241798788494
H	0.286010723428	0.290610726267	-2.213044147623
H	-1.195677956373	0.807034250095	1.803053075195
H	-2.184729631544	-0.722056386450	-2.567110246299
H	-1.975114619435	-3.798782502668	-1.905736188497
H	-0.041591420998	1.638756786497	-0.140522767135
H	-1.742298494276	-1.609051133534	2.123301675424

ENERGY = -1081.439120400000
ZPVE = 0.090404189676

(C_6H_6)-Ti^+ product

Ti	0.110108732567	-1.943691118897	-0.333150118513
C	-0.645592758131	-0.167936063253	-1.636618839464
C	-1.120178250839	-0.302251830599	0.769430852795
C	-1.494388443912	-1.231385827939	-1.863414748968
C	-2.009386968743	-1.999656761753	-0.754337947050
C	-0.266147924965	0.184555648957	-0.288508654297
C	-1.968906669135	-1.365755883655	0.542622779149
H	-0.206712266360	0.363366327673	-2.470531411246
H	-1.040940609370	0.127167289454	1.759195638275
H	-1.723150695583	-1.536667843991	-2.875730724861
H	-2.688501570518	-2.819549085206	-0.934052086113
H	0.383234580356	1.029133373687	-0.113281021337
H	-2.557392845096	-1.772866593845	1.353950123967

ENERGY = -1081.564895050000
ZPVE = 0.100697598897

$(C_2H_2)_2$-ScNC reactant

Sc	0.307062463194	-2.373199834468	0.230185285769
C	0.066091394426	-0.711583669452	2.214733529948
C	-1.162541413454	-2.887640520491	1.655588023514
C	-0.936057074243	-1.805430920861	2.441491950636
C	0.936405915339	-0.596533301592	1.180590532711
H	0.036030241385	0.037901284932	3.005998541260
H	-1.930472708991	-3.585963808186	1.969642047667
H	-1.509255726009	-1.648708162762	3.355611019953
H	1.596487780341	0.263822077742	1.171519101413
N	0.925525751811	-3.301345184846	-1.516817036536

C	1.276752866196	-3.827593090035	-2.508091076368

ENERGY = -1008.474431880000

$(C_2H_2)_3$-ScNC iNT1

Sc	-0.043582593775	-1.912635320651	0.208625706254
C	0.831172501023	-0.034999122371	0.699020011158
C	-1.244508294437	0.250917749665	-0.746284930142
C	-0.946575346611	-1.902694711794	2.706865737908
C	-1.892762533472	-2.294870549159	2.076511791458
C	-0.071149321464	0.733902945486	0.035170724052
C	-1.616673952705	-1.042634093291	-0.931394831650
H	1.642221459934	0.484037809237	1.201203285113
H	-1.809403556911	1.066558028444	-1.200249015293
H	-0.131194959094	-1.556234727887	3.298425259068
H	-2.752515177062	-2.642841041446	1.552499570732
H	0.023029089004	1.820807213494	0.020034908759
H	-2.490884507651	-1.217340972025	-1.551561676030
N	0.920430817973	-3.729949675508	-0.116485860134
C	1.415430681163	-4.791809223618	-0.204465117653

ENERGY = -1085.868191420000
ZPVE = 0.096850734417

$(C_2H_2)_3$-ScNC TS1

Sc	0.017443350412	-1.935948802219	0.310167172747
C	0.897646947143	-0.008338672902	0.614434910389
C	-1.279193225820	0.129094959358	-0.668432068741
C	-1.039405761374	-2.079321213512	2.428102454244
C	-2.063566523922	-1.862373779387	1.796861046886
C	-0.047679807701	0.692110418488	-0.061477490343
C	-1.799995837227	-1.112222526206	-0.474721659169
H	1.750584599217	0.551507778513	0.985257926765
H	-1.782490594559	0.826733623829	-1.338925506066
H	-0.380786788892	-2.321493306812	3.234083761959
H	-3.083491065566	-1.747104894250	1.512349872062
H	0.055881135405	1.763025643141	-0.240849457099
H	-2.743652413410	-1.356615465697	-0.952587198263
N	1.001751067412	-3.718088151407	-0.151542812212
C	1.539201528877	-4.747590510944	-0.328582123161

ENERGY = -1085.863465160000
ZPVE = 0.096970473682

$(C_2H_2)_3$-ScNC iNT2

Sc	0.064270483785	-2.025653973878	0.366637966873
C	0.854340539988	-0.120344325961	0.755174181917
C	-1.276074606854	-0.112127545916	-0.592858159217
C	-1.052590964721	-1.809655510342	2.130538305421
C	-2.155192408946	-1.445350718247	1.416325737776

C	-0.018475517581	0.447498698378	-0.124758193627
C	-2.184321727884	-0.916732944051	0.062208009864
H	1.701693518627	0.474099094646	1.079849173506
H	-1.590488335558	0.241134932743	-1.568227090981
H	-1.194255632816	-2.091313767626	3.168508853040
H	-3.156933495918	-1.585797612385	1.824281761704
H	0.212308424844	1.398847569657	-0.605709070890
H	-3.107858467638	-1.103094968618	-0.473839030432
N	1.167235607910	-3.714509387988	-0.178293413252
C	1.750703359810	-4.667942913008	-0.539728596727

ENERGY = -1085.932188310000
ZPVE = 0.102728974101

$(C_2H_2)_3$-ScNC TS2

Sc	-0.025317396459	-2.123358200688	-0.474293635015
C	-0.689006170987	-0.030906887262	-1.679733608138
C	-0.901811779262	-0.191273907371	0.846505025708
C	-1.600553814975	-1.065096523575	-2.100335002519
C	-1.888526123282	-2.215756063904	-1.414742759052
C	-0.381354280047	0.353945202090	-0.382002927393
C	-1.304083586670	-1.485096234545	1.049483773935
H	-0.296304907236	0.598974414991	-2.468558414617
H	-0.920871877915	0.528308570215	1.664442408630
H	-2.045143195473	-0.877760076997	-3.077282591496
H	-2.720953928428	-2.826435423617	-1.746529440757
H	0.222183594783	1.247702886393	-0.281219474924
H	-1.842055942997	-1.727659760256	1.959262749318
N	1.408279949271	-3.646834117672	-0.362572868983
C	2.252404399688	-4.462546347803	-0.320802084686

ENERGY = -1085.931971270000
ZPVE = 0.102582716915

(C_6H_6)-ScNC product

Sc	0.081046127616	-2.109766602949	-0.503268236033
C	-0.437909962761	-0.299875694083	-1.685467995862
C	-0.980211624988	-0.314183502635	0.718474348352
C	-1.629681153101	-1.119608605031	-1.894643602713
C	-2.278236255841	-1.700282092720	-0.833799874522
C	-0.331687230587	0.266542971634	-0.342363278733
C	-1.793834205398	-1.513939901884	0.532444923046
H	-0.008012646124	0.231905793037	-2.519837254858
H	-0.843097287913	0.082021100764	1.715863944389
H	-1.965442309589	-1.316204648423	-2.904186133595
H	-3.123027213271	-2.352662203474	-1.010687421885
H	0.314473084156	1.118537490689	-0.177613740864
H	-2.378289120948	-1.890419050243	1.357337140046
N	1.563987928726	-3.599896678888	-0.413227378894
C	2.391889411790	-4.431410319377	-0.363489202246

ENERGY = -1085.986438260000
ZPVE = 0.105895160876

$(C_2H_2)_2$-TiNC reactant

Ti	0.163247331898	-2.125588984227	0.941103114349
C	1.067418298065	-0.280604033595	1.793565177898
C	-1.735082947586	-2.530346752185	1.372723042516
C	-0.970688135597	-2.923218616469	2.366499966736
C	0.346965426684	0.089654794947	0.856967466399
H	1.752808989127	-0.212387337046	2.616947672039
H	-2.794244681959	-2.567264134569	1.149976261972
H	-1.129648402545	-3.422817270252	3.314095228517
H	-0.132601977385	0.756565984593	0.165871682335
N	1.197552904611	-3.289931277513	-0.314835477786
C	1.840302684689	-3.930337503681	-1.062462214970

ENERGY = -1097.160702460000

$(C_2H_2)_3$-TiNC INT1

Ti	-0.340757997938	-1.834096346571	-0.603253905940
C	0.280342993134	0.019958923992	-1.154012978215
C	-0.878558075665	0.039089436991	1.082242183262
C	-2.157887351784	-2.013282791172	-1.865883394416
C	-1.537023275083	-3.059520970536	-2.056299747928
C	-0.153165481121	0.675832505576	-0.051807037598
C	-1.195164775644	-1.275332948218	1.152971038066
H	0.801424308797	0.556322632217	-1.938302551334
H	-1.153431209825	0.732359572369	1.876733423804
H	-2.920123250983	-1.262982515334	-1.932082362205
H	-1.188597032945	-4.020301853715	-2.372934067199
H	0.003087611099	1.747482796481	0.068789899389
H	-1.726595616629	-1.662865060816	2.014204990876
N	0.999075701403	-3.314139135495	-0.577315904091
C	1.826836873065	-4.146912493818	-0.515330623134

ENERGY = -1174.605344110000
ZPVE = 0.097298935122

$(C_2H_2)_3$-TiNC TS1

Ti	-0.318952137581	-1.972546479416	-0.556400102494
C	0.102880746136	-0.067984168698	-1.162963647635
C	-0.950919025220	0.065335543363	1.074039919199
C	-1.795163584169	-1.707886444699	-2.254019673178
C	-1.925591756110	-2.825164419965	-1.732400807469
C	-0.349471154263	0.682303612930	-0.121513889141
C	-1.093427815604	-1.276041351992	1.212954461267
H	0.526877338274	0.422973698073	-2.033444237837
H	-1.271948298645	0.755879505720	1.854095316688

H	-1.947574001380	-0.868912277757	-2.899450689901
H	-2.366675622858	-3.800286696389	-1.628291683047
H	-0.298832555723	1.770102855357	-0.129352942336
H	-1.528077055106	-1.683065747674	2.119478746601
N	1.135993029470	-3.322375577358	-0.583850247363
C	2.009130032776	-4.110473231495	-0.567406653358

ENERGY = -1174.601843560000
ZPVE = 0.096779584821

$(C_2H_2)_3$-TiNC INT2

Ti	-0.209132109999	-1.999416748521	-0.424713692040
C	-0.662570943571	-0.108587783154	-1.676115220478
C	-0.900873860503	-0.197776579087	0.914673441630
C	-1.539746049450	-1.138330687726	-2.173298394824
C	-1.896846057661	-2.231299051646	-1.429227544412
C	-0.369077405196	0.275411323068	-0.356910520945
C	-1.290381202157	-1.481150417496	1.160919345602
H	-0.147725377574	0.467015150387	-2.435916822900
H	-0.873524454214	0.550864757402	1.707362772547
H	-1.823666818295	-1.027827770001	-3.222004820945
H	-2.685800293525	-2.915708383485	-1.709525879331
H	0.305132495747	1.118072725806	-0.271816793283
H	-1.768721797787	-1.777891548084	2.083518956563
N	1.224912632973	-3.393894714618	-0.500064814378
C	2.123507084754	-4.152099796096	-0.520130591206

ENERGY = -1174.653870750000
ZPVE = 0.101944757719

$(C_2H_2)_3$-TiNC TS2

Ti	-0.070757582766	-1.982461144262	-0.378317492920
C	-0.627833395960	-0.081775855964	-1.680164507329
C	-0.982652908929	-0.166421986795	0.855600272632
C	-1.490034685040	-1.133859920320	-2.108896922872
C	-1.831757488588	-2.162172857766	-1.263094923824
C	-0.373334036091	0.323873133639	-0.361187532615
C	-1.362519582193	-1.466552885815	1.054968823045
H	-0.140612718070	0.499956611872	-2.453069625255
H	-1.039425173489	0.560593149288	1.662619108607
H	-1.797084145810	-1.108177398687	-3.155519162328
H	-2.669346241861	-2.829747023738	-1.416086237716
H	0.248420684134	1.202431838942	-0.249533770331
H	-1.927684499925	-1.775872010366	1.923558327219
N	1.238102961519	-3.499085919876	-0.591180412347
C	2.093403753066	-4.304520200153	-0.628074793969

ENERGY = -1174.652896290000
ZPVE = 0.102373087908

(C_6H_6)-TiNC product

Ti	-0.172617459958	-2.001516933494	-0.423176352207
C	-0.611260709803	-0.101686736615	-1.666275574045
C	-0.834717731701	-0.195834286986	0.925661566333
C	-1.503641671424	-1.119454813211	-2.161019314870
C	-1.869138124756	-2.210287286970	-1.417892282118
C	-0.305135332785	0.274828200359	-0.347787335966
C	-1.237890338929	-1.475233492892	1.170600162798
H	-0.094281919153	0.470045789482	-2.427550108985
H	-0.793635941130	0.550063107338	1.720339242584
H	-1.792662631861	-1.002486764422	-3.207630279225
H	-2.667918402454	-2.884429110690	-1.695229946433
H	0.379519319798	1.109194436489	-0.264368191483
H	-1.714019896664	-1.769011650164	2.095289044924
N	1.244022474872	-3.412908961324	-0.511755283262
C	2.133159612057	-4.181932415028	-0.539808583081

ENERGY = -1174.653870880000
ZPVE = 0.101944449741

C_2H_2

C	0.0000000	-0.00000000	-0.59839535
C	0.0000000	0.00000000	0.59839535
H	-0.0000000	0.00000000	-1.66107013
H	-0.0000000	-0.00000000	1.66107013

ENERGY = -77.366764176100

Benzene

C	0.000000	1.390836	0.000000
C	1.204500	0.695418	0.000000
C	1.204500	-0.695418	0.000000
C	0.000000	-1.390836	0.000000
C	-1.204500	-0.695418	0.000000
C	-1.204500	0.695418	0.000000
H	0.000000	2.473947	0.000000
H	2.142501	1.236973	0.000000
H	2.142501	-1.236973	0.000000
H	0.000000	-2.473947	0.000000
H	-2.142501	-1.236973	0.000000
H	-2.142501	1.236973	0.000000

ENERGY = -232.356302845
ZPVE = 0.100180

Cartesian coordinates for all the stationary points on the potential energy surfaces obtained from the UB3LYP/def2-SVPP (GD3BJ dispersion, int=ultrafine) calculations.

$(C_2H_2)_2$-ScNC reactant

Sc	0.73206386	-2.62317335	0.78518408
C	0.79899874	-0.52505003	0.99728138
C	-1.41061001	-1.52656705	1.82408906
C	-1.20646676	-2.85579228	1.58496605
C	-0.44812082	-0.40792300	1.54194869
H	1.38311327	0.39235828	0.85337357
H	-2.35430375	-1.16840983	2.26725136
H	-2.01154046	-3.55296502	1.84829446
H	-0.85236325	0.57730348	1.82680749
N	2.09522401	-3.98198954	0.04752054
C	2.88003466	-4.76406677	-0.37626478

ENERGY = -1008.08294433
ZPVE = 0.06897423

$(C_2H_2)_3$-ScNC intermediate

Sc	0.01951004	-1.81296521	-0.42909919
C	0.39721702	0.17731404	-1.08687176
C	-1.03273045	0.19403352	1.01845147
C	-2.14287312	-2.06473875	-1.94189132
C	-1.61118697	-3.12491659	-2.20272168
C	-0.30874073	0.83838288	-0.12376069
C	-1.09146255	-1.14769036	1.26195766
H	0.88999153	0.78190867	-1.85981162
H	-1.53214513	0.91954436	1.68316444
H	-2.64642508	-1.13307149	-1.73571082
H	-1.12234275	-4.06199290	-2.42128610
H	-0.38649470	1.93910045	-0.12408964
H	-1.64997303	-1.47875066	2.14754401
N	1.12031716	-3.55992327	-0.71664440
C	1.56655084	-4.61249886	-1.02719765

ENERGY = -1085.38220221
ZPVE = 0.09729430

Transition state

Sc	0.02642658	-1.98698001	0.26620108
C	0.88519545	-0.05547589	0.59921579
C	-1.30465229	0.15010045	-0.65085507
C	-1.01753642	-1.99352304	2.42186868
C	-2.05770370	-1.84125871	1.77750006
C	-0.07150088	0.69233724	-0.03064994
C	-1.76924847	-1.13623036	-0.54123168
H	1.75558077	0.48263575	0.99813970
H	-1.85925242	0.88872889	-1.25377745

H	-0.31629015	-2.17184834	3.22563914
H	-3.08915918	-1.77157300	1.47358759
H	0.03194813	1.78444232	-0.14491199
H	-2.72478113	-1.38452308	-1.02229057
N	1.00297855	-3.77301524	-0.14292085
C	1.55024177	-4.81044188	-0.31137566

ENERGY = -1085.37736093
ZPVE = 0.09730908

C_6H_6-ScNC product

Sc	0.03102572	-2.12447558	0.21338149
C	0.29499655	-0.23789110	1.36322366
C	-1.16653868	-0.19032395	-0.63401904
C	-0.82360722	-0.84732302	2.09272742
C	-1.92433170	-1.33293730	1.41478275
C	-0.06577746	0.29521885	0.04391728
C	-1.99785129	-1.24934252	-0.04896224
H	1.11410976	0.21921703	1.92110844
H	-1.38656351	0.16862522	-1.64390698
H	-0.75160369	-0.97463596	3.17704118
H	-2.72106527	-1.84347539	1.96404920
H	0.58293000	1.03741762	-0.43089972
H	-2.91244102	-1.55703814	-0.55884138
N	1.11895378	-3.82625004	-0.33374502
C	1.73464822	-4.78934921	-0.64340618

ENERGY = -1085.50851755
ZPVE = 0.10585029

$(C_2H_2)_2$-TiNC reactant

Ti	0.17484397	-2.12737548	0.93145944
C	1.06092107	-0.27618422	1.80833250
C	-1.72079146	-2.52388032	1.35815760
C	-0.95106520	-2.91950178	2.35890134
C	0.33325121	0.09771160	0.86269510
H	1.74839800	-0.20619455	2.64753990
H	-2.79192487	-2.55290176	1.12573860
H	-1.10267146	-3.42113469	3.32200332
H	-0.15914585	0.77399048	0.16845567
N	1.19471814	-3.30252183	-0.31759244
C	1.81949594	-3.97828258	-1.06523911

ENERGY = -1096.74716275
ZPVE = 0.06344085

$(C_2H_2)_3$-TiNC intermediate

Ti	-0.30480561	-1.84491961	-0.57800644
C	0.28431439	0.00886381	-1.15627032

C	-0.92209872	0.07519196	1.05700906
C	-2.14277776	-1.96564301	-1.82960030
C	-1.54781945	-3.03644248	-2.03536416
C	-0.18880424	0.70052369	-0.07954537
C	-1.20059654	-1.25754026	1.14550723
H	0.81458261	0.53153725	-1.96109415
H	-1.24220066	0.78248131	1.83873147
H	-2.88476612	-1.17585427	-1.87335453
H	-1.21742444	-4.01455375	-2.36067955
H	-0.05910761	1.79130881	0.00518021
H	-1.74714863	-1.65313544	2.00974061
N	0.98987794	-3.36020696	-0.57671380
C	1.80048632	-4.22453317	-0.52965231

ENERGY = -1174.10473566
ZPVE = 0.09760041

Transition state

Ti	-0.30448053	-1.96516125	-0.54177284
C	0.11550621	-0.08260311	-1.18132733
C	-0.95280396	0.08038244	1.06120879
C	-1.84456161	-1.66527734	-2.19261526
C	-1.86292644	-2.84097900	-1.76344814
C	-0.33946586	0.68543191	-0.13823231
C	-1.10036052	-1.26986176	1.20973871
H	0.55431383	0.39920234	-2.06484235
H	-1.28300298	0.78562174	1.84094920
H	-2.09171511	-0.78299977	-2.76870995
H	-2.21874355	-3.86954939	-1.72816458
H	-0.27715257	1.78455517	-0.15416671
H	-1.54800199	-1.67897987	2.12388337
N	1.10140398	-3.36077841	-0.57255996
C	1.98023924	-4.15714488	-0.53846677

ENERGY = -1174.10152612
ZPVE = 0.09699607

Intermediate

Ti	-0.18093068	-2.00343823	-0.41658692
C	-0.66179182	-0.08908164	-1.68352756
C	-0.92732666	-0.17652442	0.90732192
C	-1.54885964	-1.12528278	-2.16742320
C	-1.88493713	-2.21929949	-1.40074895
C	-0.37279474	0.30127547	-0.35831406
C	-1.30773214	-1.47475909	1.13697543
H	-0.15050860	0.49669922	-2.45542513
H	-0.93434786	0.57920233	1.71066420
H	-1.86648737	-1.01514613	-3.21928160
H	-2.69217536	-2.91457142	-1.65214265
H	0.30053465	1.16004167	-0.26830543

H	-1.81757444	-1.78949372	2.05206982
N	1.22035328	-3.42453720	-0.48601823
C	2.09146343	-4.22887703	-0.48763648

ENERGY = -1174.15953081
ZPVE = 0.10213188

Transition state

Ti	-0.07119854	-1.99068463	-0.38531404
C	-0.63731482	-0.06718053	-1.68796093
C	-0.97584447	-0.16062306	0.86742103
C	-1.49956474	-1.12846049	-2.12700281
C	-1.82326505	-2.17671563	-1.28650405
C	-0.37693326	0.33661700	-0.36157283
C	-1.34091835	-1.47253315	1.06707653
H	-0.15105855	0.52930105	-2.46748957
H	-1.03701548	0.57668536	1.68243790
H	-1.82256774	-1.08601791	-3.18118818
H	-2.65769703	-2.86656165	-1.45128658
H	0.25191247	1.22581835	-0.25224713
H	-1.89591941	-1.79932450	1.95164351
N	1.23387469	-3.50234806	-0.56873050
C	2.07039522	-4.34176462	-0.58766120

ENERGY = -1174.15917632
ZPVE = 0.10237256

C_6H_6-TiNC product

Ti	-0.01033064	-1.99543846	-0.51795939
C	-0.48996145	-0.22092812	-1.64636116
C	-0.89238046	-0.37625688	0.79060734
C	-1.59929496	-1.12674636	-1.87175405
C	-2.24103830	-1.74456903	-0.78787546
C	-0.25053608	0.24146657	-0.29326635
C	-1.80165071	-1.48350093	0.56887588
H	0.01893507	0.24918977	-2.48866199
H	-0.62587454	-0.09556886	1.81267121
H	-1.86322180	-1.41003335	-2.89377025
H	-3.00275699	-2.50701782	-0.96919879
H	0.51375905	1.00132082	-0.11189587
H	-2.29048482	-1.97380703	1.41154751
N	1.40675256	-3.42240596	-0.49189873
C	2.24168038	-4.26331549	-0.47642158

ENERGY = -1174.22473953
ZPVE = 0.10575075

$(C_2H_2)_2$-MnNC reactant

Mn	0.11697491	-1.58659068	0.05277222

C	0.98222712	-0.28700454	1.41266344
C	-1.53000422	-0.58686902	-0.95980148
C	0.06667432	0.41159553	0.91453257
C	-1.38255631	-1.76576532	-1.36346847
H	1.82303915	-0.49281700	2.06783314
H	-2.00461569	0.38689604	-0.94048049
H	-0.52136841	1.31440428	0.80062464
H	-1.61174253	-2.64040628	-1.96435461
N	1.06858674	-3.26694843	0.13757610
C	1.65036628	-4.29415465	0.18958376

ENERGY = -1398.25567828
ZPVE = 0.06423274

$(C_2H_2)_3$-MnNC intermediate

Mn	-0.03313293	-1.66058296	0.21950881
C	0.93463301	-0.30535691	1.46716339
C	-1.51965328	-0.58319557	-0.97216783
C	-1.89553398	-2.33330028	2.16312244
C	-1.18759665	-3.31457004	2.08664997
C	0.07870250	0.41615373	0.90373662
C	-1.43396570	-1.78619135	-1.31334371
H	1.76024375	-0.52338745	2.13666084
H	-1.90934118	0.42564506	-1.03517095
H	-2.54130642	-1.47806269	2.25410782
H	-0.54719846	-4.17704286	2.00386010
H	-0.43185321	1.34929070	0.69855017
H	-1.67463448	-2.66971723	-1.89551127
N	1.11962621	-3.24179317	0.08118150
C	1.76695668	-4.22852492	0.05687325

ENERGY = -1475.54158384
ZPVE = 0.09826227

Transition state (TS_1)

Mn	-0.00015606	-1.75285569	0.24121908
C	0.76085104	-0.23283701	1.30305569
C	-1.36162696	-0.35222279	-0.78002581
C	-1.87402255	-2.35789415	2.15946679
C	-1.18517251	-3.35408721	2.10292354
C	-0.14555331	0.40871835	0.64730283
C	-1.37560625	-1.56974180	-1.20504938
H	1.51581585	-0.00673519	2.05477793
H	-1.85962033	0.59482776	-0.97702175
H	-2.50417070	-1.48931847	2.23390088
H	-0.56383769	-4.23224875	2.04108134
H	-0.54468371	1.41756849	0.56591074
H	-1.90530393	-2.14736617	-1.96132675
N	1.13746433	-3.30396984	0.09945681
C	1.79627587	-4.28356775	0.06083435

ENERGY = -1475.53431125
ZPVE = 0.09400919

Intermediate

Mn	-0.13418892	-1.97043777	0.38428408
C	0.88355768	-0.25632692	0.23109020
C	-1.29965815	0.45004199	-0.49235027
C	-2.03025162	-3.00482499	1.78483848
C	-1.20553014	-3.89816165	1.75548162
C	0.10829039	0.76214944	-0.19595291
C	-1.69295742	-0.82798510	-0.27932080
H	1.94848396	-0.19171102	0.48340832
H	-1.96666558	1.23663693	-0.87599567
H	-2.81046157	-2.26625604	1.85525090
H	-0.47841530	-4.69357317	1.75106831
H	0.49700656	1.78261111	-0.33367540
H	-2.72227823	-1.14436104	-0.50553465
N	1.33860136	-3.26324027	0.47243830
C	2.20282939	-4.06756729	0.52660364

ENERGY = -1475.59014033
ZPVE = 0.09826227

Transition state (TS_2)

Mn	-0.11419777	-1.95167777	0.36952190
C	0.82438240	-0.16525519	0.10685021
C	-1.39474139	0.36321426	-0.60607452
C	-1.30168597	-2.80285519	1.93706239
C	-2.03372956	-1.81232017	1.70918336
C	-0.03671021	0.79819411	-0.29936762
C	-1.79807740	-0.88841867	-0.23891521
H	1.86588605	0.05281245	0.37977781
H	-2.05559999	1.01341535	-1.20145162
H	-0.98098587	-3.65074463	2.53078865
H	-2.84925856	-1.12742063	1.89800172
H	0.24835901	1.85023704	-0.44907943
H	-2.76945469	-1.27811324	-0.56491457
N	1.29313303	-3.25642229	0.49974153
C	2.14492753	-4.07127031	0.59301424

ENERGY = -1475.57361208
ZPVE = 0.09829945

C_6H_6-MnNC product

Mn	0.21449065	-2.00744800	0.56258560
C	-0.05894726	0.03703988	1.21427680
C	-1.27509161	-0.36546837	-0.87232318
C	-0.99319915	-0.83230319	1.91902937
C	-2.09570466	-1.38088345	1.20325673

C	-0.28006782	0.30852423	-0.16637577
C	-2.19572260	-1.22206206	-0.17785283
H	0.70138782	0.59067348	1.76945881
H	-1.37935795	-0.21891491	-1.94970859
H	-0.93652830	-0.93344387	3.00503426
H	-2.82656090	-1.99766578	1.73450405
H	0.38998585	0.99526219	-0.69193750
H	-2.99395723	-1.72118639	-0.73174997
N	1.25254349	-3.55949176	0.02521705
C	1.88799843	-4.50235728	-0.29396362

ENERGY = -1475.74685784
ZPVE = 0.10630533

$(C_2H_2)_2$-CoNC reactant

Co	-0.04260718	-1.30600103	0.03964951
C	1.10079441	-0.30991625	1.37077461
C	-1.41572451	-0.62658730	-1.29173142
C	0.03415196	0.32754244	1.24141967
C	-1.42538173	-1.87560096	-1.31523881
H	2.07792556	-0.63544365	1.70413061
H	-1.69564780	0.38483510	-1.56004371
H	-0.69262449	1.11301004	1.40928268
H	-1.64371827	-2.90418859	-1.57247531
N	0.89222814	-2.97382386	0.13239966
C	1.46818528	-4.00148602	0.18931334

ENERGY = -1629.99595408
ZPVE = 0.06441093

$(C_2H_2)_3$-CoNC intermediate

Co	-0.21355403	-1.75019007	0.42138055
C	1.14968785	-0.34673308	1.20470635
C	-1.72733841	-0.88639332	-0.85268511
C	-1.90643953	-2.06185933	2.02987105
C	-1.23304985	-3.08107577	1.99869825
C	0.05550609	0.22936748	1.24030277
C	-1.13218161	-1.77453825	-1.47511679
H	2.18612204	-0.64588566	1.26362735
H	-2.41981409	-0.08535183	-0.63664112
H	-2.60098994	-1.25609540	2.19279021
H	-0.69476055	-4.01424225	2.03691772
H	-0.68201838	1.00235017	1.40282632
H	-0.75849173	-2.48809555	-2.19508812
N	1.04603089	-3.16137340	0.10123707
C	1.81494307	-4.03417477	-0.08780547

ENERGY = -1707.27808022
ZPVE = 0.09319743

Transition state (TS_1)

Co	-0.13574784	-1.84508614	0.40599315
C	0.73270479	-0.30532729	1.26222017
C	-1.37057320	-0.37937186	-0.72000621
C	-1.84285730	-2.23441426	2.06795020
C	-1.17165885	-3.24862996	2.03474916
C	-0.18701902	0.36151202	0.66954368
C	-1.31559308	-1.58758126	-1.14313177
H	1.57042272	-0.19142152	1.94558668
H	-1.91141583	0.54219029	-0.92792816
H	-2.48683386	-1.37773798	2.16166316
H	-0.59854147	-4.16164182	2.03151568
H	-0.57744701	1.37717684	0.63772764
H	-1.71320873	-2.24700052	-1.91046756
N	1.07216600	-3.23645342	0.11904037
C	1.82625575	-4.12794336	-0.04794987

ENERGY = -1707.25063252
ZPVE = 0.09391151

Intermediate

Co	-0.09901912	-2.16534702	-0.57029313
C	-0.02634187	-0.43701559	-1.49451437
C	-0.59248334	0.27346471	0.68575129
C	-0.95257359	-3.80314351	-2.04971977
C	-1.24968075	-2.74359925	-2.57598683
C	-0.27760406	0.62987116	-0.69511673
C	-0.55395928	-1.05219475	0.93453315
H	0.24855755	-0.31503200	-2.55312265
H	-0.81354847	1.02396191	1.45757461
H	-0.73033367	-4.76712361	-1.62204795
H	-1.57767296	-1.89198537	-3.14612545
H	-0.25117883	1.67503029	-1.03370620
H	-0.72183072	-1.55776751	1.89172269
N	0.36921740	-3.71201321	0.43425516
C	0.66053659	-4.67059749	1.05800441

ENERGY = -1707.31587335
ZPVE = 0.09902157

Transition state (TS_2)

Co	-0.08319857	-2.36271230	0.71846991
C	0.57257562	-0.56598393	1.34963393
C	-0.97780219	-0.16274555	-0.46824776
C	-1.87947031	-2.87969946	1.61883981
C	-1.13261337	-2.35233992	2.46455024
C	-0.04529730	0.33760857	0.56054760
C	-1.22439783	-1.47043264	-0.59472577
H	1.25864424	-0.26158725	2.15129132

H	-1.48038195	0.54585482	-1.14541016
H	-2.70337588	-3.37388372	1.12379039
H	-0.74984341	-1.97679987	3.40400690
H	0.11110142	1.42409582	0.62708108
H	-1.81954689	-2.05203356	-1.30008881
N	0.76065495	-3.42269795	-0.54868230
C	1.28360456	-4.08837329	-1.37455008

ENERGY = -1707.29187399
ZPVE = 0.09718975

C_6H_6-CoNC product

Co	0.11408304	-2.24065660	0.38107575
C	0.00586665	-0.74047160	1.97374931
C	-1.03708116	-0.44415937	-0.22510602
C	-1.90008899	-2.13671806	1.31997264
C	-0.91699054	-1.77259967	2.26992010
C	-0.05354413	-0.07734538	0.72418239
C	-1.96032082	-1.47200110	0.07408186
H	0.78540927	-0.48120386	2.69353554
H	-1.05621943	0.03860403	-1.20453327
H	-2.58366701	-2.96158888	1.53190864
H	-0.84700245	-2.31173557	3.21722040
H	0.68154820	0.69250374	0.47929725
H	-2.68823731	-1.78628140	-0.67682910
N	0.93266307	-3.64731715	-0.59064646
C	1.52796891	-4.50053426	-1.14772865

ENERGY = -1707.50659102
ZPVE = 0.10868226

$(C_2H_2)_2$-NiNC reactant

Ni	-0.17871135	-2.01526477	1.01229742
C	0.92437226	-0.27784366	2.03184117
C	-1.52720606	-3.24465864	1.59966064
C	-1.17039048	-2.57688740	2.60872871
C	1.47450642	-0.42564547	0.94901267
H	0.57212457	-0.00138687	3.01158062
H	-2.05229596	-3.98762610	1.00868886
H	-1.20393182	-2.32573364	3.66393933
H	1.99725127	-0.49078361	0.00782945
N	0.24796950	-2.42953064	-0.78525077
C	0.52234115	-2.66091431	-1.90787617

ENERGY = -1755.52433859
ZPVE = 0.06554124

$(C_2H_2)_3$-NiNC intermediate

Ni	-0.32813062	-1.57440824	0.38517805

C	1.19518881	-0.77739116	1.52748809
C	-1.88619458	-0.54826110	-0.57763729
C	-1.23593177	-3.23748220	1.95310441
C	-1.81638798	-2.21442843	2.25687256
C	0.34716021	0.11931953	1.42573395
C	-1.74730752	-1.65706956	-1.11135201
H	2.06052798	-1.38931112	1.73585457
H	-2.26079464	0.44429862	-0.37317665
H	-0.72471351	-4.14755708	1.68838413
H	-2.37230098	-1.35603694	2.58837423
H	-0.13899670	1.07846017	1.53063091
H	-1.80021536	-2.54358004	-1.72614722
N	0.66102865	-3.12169082	-0.20210250
C	1.25525305	-4.07728141	-0.54622563

ENERGY = -1832.81184233
ZPVE = 0.09317026

Transition state (TS_1)

Ni	-0.45524626	-1.81327980	0.60226645
C	0.72361434	-0.51459570	1.42553561
C	-1.17961092	-0.22526581	-0.70032871
C	-1.87486721	-3.01234675	1.63364091
C	-1.01579568	-2.68917823	2.45579518
C	0.15551611	0.27704987	0.57764120
C	-1.66325529	-1.41243848	-0.85859797
H	1.50390634	-0.47706444	2.18181230
H	-1.31053913	0.74022289	-1.18703576
H	-2.70847998	-3.44065743	1.10059633
H	-0.38661306	-2.56713399	3.32282039
H	0.25129430	1.32802632	0.30843664
H	-2.35867898	-1.93025292	-1.51469786
N	0.73489928	-3.06801640	-0.14895020
C	1.47450922	-3.85679935	-0.61242820

ENERGY = -1832.77233916
ZPVE = 0.09398538

Intermediate

Ni	-0.06494007	-1.96737136	-0.61295914
C	-0.01886379	-0.21921997	-1.45839512
C	-1.00631523	0.35572577	0.58936947
C	-1.40087868	-3.42514836	-1.89243669
C	-1.76426234	-2.30135435	-2.19858527
C	-0.47965251	0.79895918	-0.70618474
C	-0.90593765	-0.96570197	0.79690061
H	0.41268927	-0.11121050	-2.46581712
H	-1.45002625	1.03625143	1.32960061
H	-1.13132533	-4.43464142	-1.62805929
H	-2.17174333	-1.36669188	-2.54393407

H	-0.47074897	1.85324501	-1.01235909
H	-1.23827204	-1.58342917	1.63717587
N	0.42951091	-3.53920367	0.30465597
C	0.75447927	-4.52930981	0.85815384

ENERGY = -1832.84620930
ZPVE = 0.09949707

Transition state (TS_2)

Ni	-0.21256517	-2.48342921	-0.71945376
C	0.54723265	-0.72688960	-1.33280021
C	-0.94608882	-0.20690924	0.46261134
C	-1.89085212	-2.94836083	-1.73250219
C	-1.23766201	-2.14963527	-2.43523499
C	0.01353210	0.23128833	-0.55339698
C	-1.21300599	-1.51069759	0.57616140
H	1.28193446	-0.55543106	-2.12965860
H	-1.44444307	0.51460862	1.12609827
H	-2.66861397	-3.62111405	-1.39493479
H	-0.98025066	-1.52810857	-3.28128206
H	0.28375635	1.29408605	-0.62160569
H	-1.80990288	-2.08452343	1.28669530
N	0.76835495	-3.20299420	0.64326375
C	1.39922727	-3.68362017	1.51953292

ENERGY = -1832.82804112
ZPVE = 0.09809506

C_6H_6-NiNC product

Ni	-0.06099470	-2.64362905	0.43902943
C	0.03357077	-0.67125889	1.87293335
C	-1.27575236	-0.14460408	-0.10195473
C	-1.89242518	-2.06112553	1.29880241
C	-0.82859563	-1.74975083	2.18265998
C	-0.22754974	0.16191061	0.76843969
C	-2.06733724	-1.28595614	0.12802355
H	0.88001990	-0.45628648	2.53012044
H	-1.45822738	0.47218554	-0.98535078
H	-2.58479825	-2.87551009	1.52645138
H	-0.68596678	-2.31939030	3.10439598
H	0.41886166	1.02109451	0.57329606
H	-2.86000840	-1.55082212	-0.57632859
N	1.11760639	-3.53574326	-0.66509503
C	1.87115351	-4.10513430	-1.37117329

ENERGY = -1833.03847402
ZPVE = 0.10814122

$(C_2H_2)_2$-$Sc^+(H_2O)_8$ reactant

Sc	-0.02283275	-1.27068498	-0.26782161
C	-1.75850524	-2.46496433	-0.62959776
C	-1.56545822	-2.02523749	1.84848974
C	-0.35463109	-1.39731782	1.81471854
C	-2.28122991	-2.51297470	0.63422048
H	-2.37126265	-2.85006234	-1.45624745
H	-2.09462094	-2.20198891	2.79793731
H	0.09700273	-1.08700732	2.76609509
H	-3.28672744	-2.92212985	0.82634357
H	3.93307859	-5.82357257	-0.94636295
H	3.79989273	-7.42821843	-0.67984107
H	4.41624655	-3.83980132	-0.51117204
H	3.98900311	-4.02880261	-2.00184444
H	2.80168134	-3.93319184	1.06012980
H	3.69316962	-2.60769743	1.55816930
H	2.43191352	-2.34504300	-0.11173802
H	1.95299079	-2.57631605	-1.72157514
H	1.85237412	-4.44582476	-2.56457339
H	2.32122721	-3.28922918	-3.68160360
H	1.99210489	-6.23838113	-1.71894993
H	0.56317979	-5.58475249	-1.48422188
H	2.55592123	-5.87646404	0.67455971
H	1.15622383	-5.16634931	0.93241960
H	-0.88398679	-4.33856652	-0.10281457
H	-1.03820522	-5.85850410	0.27281484
O	3.30364122	-6.59150610	-0.68572754
O	4.54027291	-4.42883891	-1.29034397
O	3.31081000	-3.04356484	0.77663898
O	1.71368101	-2.03601640	-0.83724866
O	2.34349609	-3.51419285	-2.73512939
O	1.28248766	-5.68565422	-2.15227395
O	2.10025546	-5.17358996	1.21728549
O	-0.39008463	-5.18841344	-0.01348410

ENERGY = -1526.08933270
ZPVE = 0.26158457

Intermediate

Sc	-0.10290986	-1.17248980	-0.19171128
C	-1.83201218	-2.24269005	-0.87967959
C	-1.96999883	-1.96935885	1.62882195
C	-1.11479868	1.11480941	-1.11518351
C	-0.70815619	1.41749213	-0.01009183
C	-2.53324324	-2.31601219	0.29603045
C	-0.72740890	-1.43205288	1.81884923
H	-2.36471971	-2.52355442	-1.79947814
H	-2.64533886	-2.16834518	2.47601119
H	-1.50854696	0.91161269	-2.10064558
H	-0.37552046	1.74114862	0.96565097
H	-3.58674792	-2.64061883	0.31716027

H	-0.41670105	-1.22755127	2.85247608
H	3.83359376	-5.67361498	-0.98205228
H	3.63182341	-7.29124312	-0.90765242
H	4.33048798	-3.77587840	-0.28336048
H	4.04684755	-3.78669264	-1.82047167
H	2.58909579	-3.96436216	1.11438084
H	3.48385704	-2.75932372	1.83932840
H	2.36350455	-2.24328366	0.07651955
H	2.04607599	-2.28093177	-1.56870257
H	1.98395109	-4.04948841	-2.64767797
H	2.61811205	-2.81963470	-3.57938941
H	1.96859953	-5.93132172	-1.98767193
H	0.54673311	-5.24696441	-1.82256697
H	2.29834044	-5.84440650	0.48273506
H	0.91126930	-5.09319235	0.67070813
H	-1.00019385	-4.08542647	-0.48618232
H	-1.21804028	-5.62402459	-0.23713198
O	3.16018095	-6.44135375	-0.87132834
O	4.51740272	-4.28038119	-1.10887406
O	3.15599845	-3.08098244	0.98177817
O	1.72463765	-1.85490318	-0.66424311
O	2.52203554	-3.13499240	-2.66419939
O	1.32365074	-5.31016789	-2.42782506
O	1.81464954	-5.18584002	1.05488853
O	-0.53190220	-4.95457281	-0.41580391

ENERGY = -1603.39466345
ZPVE = 0.29040540

Transition state

Sc	-0.09701002	-1.15404014	-0.34728693
C	-1.86266108	-2.25135380	-0.94978209
C	-1.93750975	-1.84876538	1.53172081
C	-0.84234986	1.04687836	-0.72207898
C	-1.04155170	1.09180575	0.49892651
C	-2.51937338	-2.31575474	0.24915811
C	-0.85050684	-1.02119588	1.64972742
H	-2.39964860	-2.62109868	-1.83456512
H	-2.45266285	-2.21828960	2.43321856
H	-0.80329209	1.45663684	-1.72742274
H	-1.26123720	1.40774437	1.50751306
H	-3.53474774	-2.73639687	0.33881127
H	-0.54004691	-0.71170450	2.65616339
H	3.83988563	-5.70010771	-0.94619537
H	3.63552610	-7.31367962	-0.81076274
H	4.34521965	-3.77894606	-0.32859919
H	4.05779609	-3.85026324	-1.86393074
H	2.63172263	-3.90394855	1.10287257
H	3.56386474	-2.69532838	1.76601287
H	2.39565599	-2.21591824	-0.01494852

H	2.06694051	-2.32683175	-1.64737421
H	1.98674374	-4.12878352	-2.66134755
H	2.61504570	-2.94358196	-3.64913344
H	1.96366333	-5.98627892	-1.91682932
H	0.54653136	-5.28959576	-1.76475726
H	2.32373113	-5.81365419	0.54299566
H	0.93634329	-5.06290429	0.71349577
H	-1.04895084	-4.13783153	-0.44084037
H	-1.19357773	-5.68056845	-0.17027001
O	3.16628154	-6.46176337	-0.79726650
O	4.52669269	-4.31752070	-1.13365434
O	3.19760018	-3.03309480	0.93057060
O	1.74789765	-1.87160046	-0.75862037
O	2.53008929	-3.22278940	-2.72110585
O	1.31706340	-5.37887378	-2.37332520
O	1.84265614	-5.13413974	1.09275373
O	-0.54033904	-4.97958301	-0.35208775

ENERGY = -1603.38853118
ZPVE = 0.29065139

C_6H_6-$Sc^+(H_2O)_8$ product

Sc	-0.11352771	-1.87636362	0.46840164
C	-1.52598625	-2.19150641	-1.20703995
C	-2.40046512	-1.03104515	0.79366473
C	-0.93380229	-0.90377799	-1.60960496
C	-0.84979199	0.13682146	-0.71152335
C	-2.49473078	-2.06932446	-0.10527957
C	-1.34017803	-0.02324818	0.65968503
H	-1.71273045	-2.94353620	-1.97772480
H	-3.08064911	-0.98205897	1.64954572
H	-0.49506995	-0.80284304	-2.60702026
H	-0.33957181	1.06325443	-0.99146958
H	-3.25143931	-2.84781870	0.03159680
H	-1.33800631	0.83958240	1.32818249
H	3.52485710	-5.67896061	-1.69842921
H	3.10205679	-7.14229307	-2.26197155
H	4.45624839	-4.11361918	-0.55099224
H	4.03195910	-3.59640366	-1.96520610
H	2.91095484	-4.36331153	0.95360278
H	4.04850290	-3.76907098	1.93214207
H	2.69265933	-2.38181261	0.58952995
H	2.28094052	-1.86484269	-0.87656213
H	1.97693045	-3.10143874	-2.55846775
H	2.71651623	-1.80186977	-3.17144807
H	1.64505648	-5.33745559	-2.36802782
H	0.19254761	-4.62521482	-2.59294087
H	1.85815929	-6.04892185	0.01241055
H	1.28678949	-4.73329660	-0.54666801
H	-0.72662480	-3.97485793	2.32295410

H	0.49233236	-4.44867315	1.37025914
O	2.76515980	-6.36035410	-1.79200858
O	4.38892183	-4.38396338	-1.49476719
O	3.62723717	-3.67550487	1.05955773
O	1.98235206	-1.88690343	0.08470525
O	2.65426424	-2.38211078	-2.39278155
O	1.09768729	-4.48729718	-2.26335029
O	1.54017545	-5.16797350	0.31774537
O	-0.01666857	-3.66357234	1.73575333

ENERGY = -1603.54925997
ZPVE = 0.30150535

$(C_2H_2)_2$-$Ti^+(H_2O)_8$ reactant

H	3.85784485	-5.08351557	-1.93500175
H	3.96706963	-6.51229444	-2.71753723
H	4.21127392	-3.66583282	-0.45059911
H	3.41771264	-3.06698589	-1.65430477
H	3.19036175	-5.06576218	0.96879722
H	4.06228414	-4.06608961	1.98434066
H	2.29614045	-3.21628342	1.11561119
H	1.43984355	-2.57099967	-0.18225738
H	1.26547829	-3.61434737	-1.94936944
H	1.27951619	-1.96810583	-2.23306050
H	1.81955441	-5.48224450	-2.40080843
H	0.48442592	-5.46673045	-1.53958087
H	3.08320486	-6.43527286	-0.48453855
H	1.76907901	-6.43512940	0.40853930
H	-0.44560922	-5.61040866	0.64144246
H	-0.54198137	-7.04122393	-0.00637599
O	3.41944684	-5.99717647	-2.10020160
O	4.18576148	-3.64352171	-1.43510047
O	3.46735330	-4.07111377	1.21449138
O	1.40618044	-2.70708345	0.86417283
O	1.59423750	-2.65896598	-1.62453577
O	0.96489334	-4.98767961	-2.25425999
O	2.74830137	-6.33256648	0.45023088
O	0.00568917	-6.23535471	0.03268772
Ti	-0.22185173	-2.68777438	1.94862996
C	-0.58885772	-1.29601706	3.68905924
C	-1.73038778	-3.90015738	1.48106218
C	-1.05420157	-4.40898593	2.50420241
C	-1.22116427	-0.82008106	2.73215080
H	-0.16721140	-1.45208333	4.67820275
H	-2.62454231	-4.17647113	0.90775098
H	-1.14241207	-5.29174459	3.15034151
H	-1.85538168	-0.18145007	2.12349140

ENERGY = -1614.75315261

ZPVE = 0.25646245

Intermediate

Ti	0.24991475	-0.67677797	-0.77838593
C	-0.62269736	-0.67653368	1.07260172
C	-2.31165108	-1.60362153	-0.48264003
C	0.21615235	1.36370294	-1.53259411
C	-0.67699082	0.85826080	-2.24262787
C	-1.89263023	-1.13124827	0.84817460
C	-1.41938827	-1.61408652	-1.53135234
H	-0.33466557	-0.33071342	2.07413456
H	-3.36495726	-1.89949564	-0.60392014
H	0.85315915	2.17796678	-1.18697330
H	-1.46978674	0.71771444	-2.96558159
H	-2.64980602	-1.15567134	1.64672713
H	-1.76770932	-1.93537908	-2.52310921
O	2.91215456	-6.30405971	-0.73650026
O	4.47738028	-4.29874151	-1.05057168
O	3.06866327	-2.82426776	0.83979759
O	1.89331412	-1.63812871	-0.97525240
O	2.68701524	-3.10044932	-2.81005356
O	1.27473731	-5.11771921	-2.46188900
O	1.59967793	-4.81187675	1.02784906
O	-0.63461265	-4.46624924	-0.56843898
H	3.66122270	-5.61372961	-0.86435264
H	3.29904479	-7.19548517	-0.68982666
H	4.29063813	-3.71016292	-0.28408672
H	4.11580170	-3.82291412	-1.83335220
H	2.43222195	-3.66719248	1.01842851
H	3.36302186	-2.42895597	1.67908340
H	2.43357969	-2.04416686	-0.13302868
H	2.23765186	-2.16347229	-1.83898153
H	2.05981799	-3.96072769	-2.75341296
H	2.82995632	-2.84009538	-3.73678985
H	1.83395962	-5.76593164	-1.94858473
H	0.48309969	-4.93378939	-1.90221246
H	2.05203606	-5.54717516	0.52516676
H	0.71909685	-4.67958882	0.60236558
H	-0.98927969	-3.55522166	-0.69784353
H	-1.40514501	-5.04703324	-0.42729431

ENERGY = -1692.11152348
ZPVE = 0.28893764

Transition state

Ti	0.21405218	-0.84737778	-0.88057264
C	-0.52388953	-1.02040057	0.96802999
C	-2.31105095	-1.37941042	-0.70118190
C	-0.21045010	1.69267051	-0.83288220
C	-0.60548071	1.37805542	-1.94065903

C	-1.86837737	-1.03945063	0.64428988
C	-1.40231178	-1.74699774	-1.68275061
H	-0.21732034	-0.84396386	2.00830271
H	-3.38777164	-1.28410048	-0.90841243
H	0.11397373	2.06663435	0.12691056
H	-0.97492775	1.18286827	-2.93601105
H	-2.64840242	-0.76289161	1.36943327
H	-1.77454790	-2.04943225	-2.67199308
O	2.92167996	-6.30949551	-0.53851610
O	4.49412773	-4.35761956	-1.08027778
O	3.17359733	-2.70366410	0.70912930
O	1.92690290	-1.66575823	-1.15376951
O	2.63965470	-3.30512338	-2.86891644
O	1.25760475	-5.30089623	-2.34943955
O	1.65897012	-4.62442556	1.09120305
O	-0.54989737	-4.35068320	-0.52644928
H	3.67324933	-5.64279995	-0.74840980
H	3.30213913	-7.19552824	-0.41005501
H	4.33635682	-3.70352206	-0.36122841
H	4.09893788	-3.94992624	-1.88507016
H	2.51940986	-3.51388950	0.97186119
H	3.49614781	-2.24765704	1.50580913
H	2.50678259	-1.99711248	-0.30921773
H	2.22810824	-2.27750246	-1.97816296
H	2.01979149	-4.16769432	-2.72814980
H	2.76782486	-3.12951298	-3.81735495
H	1.82512049	-5.89408110	-1.78131734
H	0.47018706	-5.06605100	-1.80007868
H	2.08724358	-5.41546576	0.65829699
H	0.78336715	-4.50085113	0.64861153
H	-0.77625312	-3.42370405	-0.80608184
H	-1.38929781	-4.77433845	-0.26917414

ENERGY = -1692.10321753
ZPVE = 0.28883045

C_6H_6-$Ti^+(H_2O)_8$ product

Ti	0.21548960	-0.97518701	-0.88117060
C	-0.94298971	0.06816242	0.76813090
C	-1.90623483	-1.45202241	-0.93519474
C	-0.50781477	0.97064602	-0.28024379
C	-1.04628223	0.78148593	-1.60444326
C	-1.63930252	-1.10195716	0.44940431
C	-1.69681161	-0.41168565	-1.93830107
H	-0.65791682	0.26875742	1.80395290
H	-2.57730764	-2.28111258	-1.17242882
H	0.03022136	1.88553096	-0.02778856
H	-0.82816354	1.51647144	-2.38395311
H	-1.89139456	-1.81337546	1.24069105
H	-2.01553940	-0.59362727	-2.96779623

O	2.93953966	-6.34511134	-0.45793318
O	4.54511453	-4.46451089	-1.12509587
O	3.38274365	-2.69037395	0.66409179
O	1.95852162	-1.80816452	-1.18656187
O	2.67439288	-3.44500772	-2.91739288
O	1.26445359	-5.40505066	-2.29032230
O	1.76623763	-4.54966131	1.11244003
O	-0.52977855	-4.25900892	-0.49022969
H	3.70024838	-5.70466346	-0.71669728
H	3.30519005	-7.23229088	-0.29713281
H	4.44293291	-3.77194207	-0.43248473
H	4.14649612	-4.07646174	-1.93756842
H	2.73082697	-3.45230286	0.97947668
H	3.78523466	-2.23476780	1.42377087
H	2.61927042	-2.02704950	-0.40014505
H	2.26231623	-2.40816488	-2.00614008
H	2.05850569	-4.29195878	-2.75280696
H	2.79377866	-3.28601017	-3.87005219
H	1.83088602	-5.97651115	-1.69814078
H	0.49724914	-5.11668164	-1.74512147
H	2.16443882	-5.37362655	0.71276424
H	0.91262563	-4.40234665	0.64635064
H	-0.88067266	-3.39615307	-0.82722159
H	-1.29542590	-4.75606109	-0.14555632

ENERGY = -1692.22672126
ZPVE = 0.29900533

C_2H_2

C	0.00000000	0.00000000	-0.60451311
C	0.00000000	0.00000000	0.60451311
H	0.00000000	0.00000000	-1.67998283
H	0.00000000	0.00000000	1.67998283

ENERGY = -77.26885621
ZPVE = 0.02377147
